# Odorant and Taste Receptors in Sperm Chemotaxis and Cryopreservation: Roles and Implications in Sperm Capacitation, Motility and Fertility

**DOI:** 10.3390/genes12040488

**Published:** 2021-03-27

**Authors:** Malik Ahsan Ali, Yihan Wang, Ziyue Qin, Xiang Yuan, Yan Zhang, Changjun Zeng

**Affiliations:** 1 College of Animal Science and Technology and Farm Animal Genetic Resources Exploration and Innovation Key Laboratory of Sichuan Province, Sichuan Agricultural University, Chengdu 611130, Sichuan, China; sonic123wang@163.com (Y.W.); Qinziyue1234@163.com (Z.Q.); yxmercury@163.com (X.Y.); yanzhang@sicau.edu.cn (Y.Z.); 2 Department of Theriogenology, Riphah College of Veterinary Sciences, Lahore 54000, Punjab, Pakistan; malik364ahsan@hotmail.com; 3 Department of Theriogenology, Faculty of Veterinary Science, University of Agriculture, Faisalabad 38000, Punjab, Pakistan

**Keywords:** sperm chemotaxis, odorant receptor, taste receptor, olfactory transduction, mammalian sperm, post-thaw sperm motility

## Abstract

Sperm chemotaxis, which guide sperm toward oocyte, is tightly associated with sperm capacitation, motility, and fertility. However, the molecular mechanism of sperm chemotaxis is not known. Reproductive odorant and taste receptors, belong to G-protein-coupled receptors (GPCR) super-family, cause an increase in intracellular Ca^2+^ concentration which is pre-requisite for sperm capacitation and acrosomal reaction, and result in sperm hyperpolarization and increase motility through activation of Ca^2+^-dependent Cl^¯^ channels. Recently, odorant receptors (ORs) in olfactory transduction pathway were thought to be associated with post-thaw sperm motility, freeze tolerance or freezability and cryo-capacitation-like change during cryopreservation. Investigation of the roles of odorant and taste receptors (TRs) is important for our understanding of the freeze tolerance or freezability mechanism and improve the motility and fertility of post-thaw sperm. Here, we reviewed the roles, mode of action, impact of odorant and taste receptors on sperm chemotaxis and post-thaw sperm quality.

## 1. Introduction

All organisms are dependent on odorant and taste receptors for survival as these are primarily involved in detecting diverse environmental cues like, finding and selection of food, avoiding life threatening predators and pollutants. The ORs and TRs are primarily expressed in nasal and oral sensory cells and respond only to their specific ligands [1,2] and convey electrical signals to higher perception centers.

These ORs and TRs are not only limited to nasal and oral tissues but are also expressed in ectopic organs which indicates their diverse biological functions. However, the concept of chemotaxis was originated from sea animals as they release their sperm and egg in the environment for fertilization, but the role of chemotaxis via ORs in mammals’ sperm and egg communication was established in late 1900s [3,4]. Moreover, discovery of ORs and TRs in sperm, prostate gland, and testes of human [5,6,7,8,9,10] have strengthen the belief about their role in sperm chemotaxis and reproduction success. It was reported that OR4C13, MOR23, OR1E2, and OD1 have been localized in testes of human [10], mouse [11], dog [12], and rat [13] respectively. Similarly, TAS2R3, TAS2R124, and α-gustducin have been localized in testes of human [14], mouse [15], and pig [16]. These ORs and TRs can modulate the motility, fertility, and survivability of sperm, and are even involved in gametogenesis at mitotic and meiotic levels [14]. Egg releases chemicals to attract sperm cell and existence of chemoattractant gradient give cues about interaction of sperm-egg through chemotaxis [4]. Additionally, chemotaxis in mammals is more complicated compared to sea animals as sperm movement in mammals is also dependent on thermal gradient along with chemicals released from the fallopian tube [17,18], oocyte, and cumulus oophorous cells [19]. Binding of these chemicals with their receptors present on sperm stimulate the voltage and ion gated channels, which induces capacitation like changes and cause hypermotility [20,21].

Recently, Ran and colleagues [22,23] reported that olfactory transduction pathway in pig and giant panda maybe associated with sperm freeze tolerance or freezability, and the mRNA expression level of some odorant receptors were dysregulated during cryopreservation. These findings indicated that ORs and TRs maybe associated with post-thaw sperm motility, acrosomal reaction, cryo-capacitation-like changes, and fertility. Although studies on ORs and TRs have already achieved some milestones, but only few manuscripts are available regarding their role in sperm chemotaxis and survival during and after cryopreservation. In one study, we have determined the differential expression of piRNA (related to olfactory transduction pathway) in fresh and frozen thawed pig and giant panda sperm (unpublished work) which indicate substantial future scope of this subject in improving artificial breeding. This review summarizes the classification and distribution of ORs and TRs, and regulating sperm chemotaxis, capacitation, motility, and fertility via ion and voltage gated channels in sperm. In addition, the challenges, limitations and future directions in sperm cryoinjury, freeze tolerance, or freezability during cryopreservation are also included.

## 2. Sperm Chemotaxis and Sperm–Egg Interaction

Currently, three guidance mechanisms are known for the sperm movement including thermotaxis (swimming due to temperature gradient), Rheotaxis (swimming due to fluid flow gradient), and chemotaxis (swimming due to concentration difference of chemoattractants) which have been demonstrated in human, rabbit, mice, bull and pig [3,17,24,25,26,27,28,29,30]. How chemotaxis occur in sperm through the involvement of ORs and TRs is the focus of this review as expression or suppression of these receptors effect motility, fertility, and viability of post-thaw sperm.

Mammalian sperm chemotaxis is a recent subject relative to other guidance mechanisms of sperm as it is based on the competitive nature of sperm in the female reproductive tract to reach the site of fertilization [20]. In fact, millions of sperm are ejaculated during natural mating but only fewer reach the egg to fertilize it [31]. Sperm maturation requires capacitation which leads to hyperactivation and enables them to penetrate viscoelastic environment in oviduct and finally cumulus oophorous cells surrounding the egg in mouse and pig [32,33]. This penetration results in acrosomal reaction in sperm and help in fusion with egg which results in fertilization [34]. Furthermore, chemo-attraction occurs only in capacitated sperm with unreacted acrosome and not in sperm with reacted acrosome as described in different mammalian species like human, mouse, bull, and rabbit [34,35,36,37,38] with the help of sperm selection assay (SSA) which suggest disorientation of sperm in reproductive tract due to premature acrosomal reaction [34].

Additionally, role of sperm chemotaxis in mammals is to select a group of capacitated sperm to guide toward egg but in case of non-mammalian species is to attract as much sperm as possible near the egg [39]. During copulation or artificial insemination a large number of sperm are ejaculated/deposited in the female reproductive tract and they get stored (in isthmus) in the tract to wait for the egg to be released [40,41]. At the time of ovulation only capacitated and hyperactivated sperm move toward the egg, possibly under some chemical stimulation released from the egg [39,42]. Moreover, observations in rats, mice, and pigs further strengthen the role of chemoattractants in sperm motility and fertilization capability as only single sperm is guided to one egg and after penetration of egg walls chemical signal stops and impedes the movement of other sperms toward egg [43].

Several studies have reported chemoattraction of human, mouse, pig, and rabbit sperms toward the gradient of progesterone (P4) secreted from cumulus oophorous cells of egg near ovulation [36,44,45,46,47]. This movement of sperm is due to mobilization of Ca^2+^ from cytoplasm preceded by membrane channels activation [48]. Membrane channels participating in regulation of Ca^2+^ movement in spermatic cell includes CatSper [49,50], cyclic nucleotide gated (CNG) [51,52], and calcium voltage-gated channels (CaV) [53,54]. These channels have been localized in head, mid piece, and more abundantly in tail region of human, pig, and other mammals’ sperm [55,56,57], which suggest capacitation and hypermotility are dependent on activation of these channels (Table 1). Moreover, the co-existence of ORs, TRs, and membrane channels indicate their coordinated functionality. The currently known molecular mechanism and role of ORs and TRs in chemotaxis of sperm are discussed in Section 4 and Section 5 of this review.

## 3. Odorant and Taste Receptors

Buck and Axel [74] discovered the genes of olfactory system in rat which is considered as the beginning of molecular research on olfactory transduction and ORs. It is assumed that the odorant receptors play their role in organ construction and cell–cell identification as ORs develop in spleen, heart, and chicken notochord [75,76]. The proposed function of these embryonic ORs is to position the somatic cells and neural tube. The ORs have been classified into two major classes based on their distinct amino acid arrangements and distribution. The class I ORs were first discovered in fish [77] and then in frog [78], and later it was determined that fishes including goldfish and teleost fish have only class I odorant receptor genes [79]. At the beginning, it was believed that only class II ORs exist in mammals and they lack class I receptors [77]. Moreover, scientists also believed that class II ORs are meant to detect volatile odorants and class I for water-soluble odorants. But, the recent genomic analysis contradicts this believe and proved that human and mouse genome have many class I OR genes [80,81,82,83,84]. Coelacanth, an evolutionary link between fish and tetrapod has many class I OR genes along with 7 class II OR genes [85]. The majority of ORs in mammals are class I receptors and it indicates that class I receptors play a crucial role in the olfaction [80,86]. The duplication and pseudogenization of genes were more in class II than class I genes as reported by Niimura et al., [87] by using reconciled-tree method.

Similar to ORs, TRs were first characterized in early 2000s [88] which resulted in discovery of six types of TRs till today. Out of these six types (salty, bitter, sweet, umami, sour, and fat) sour and salty taste sensations are carried out via membrane ion channels while the rest of them are governed by GPCRs like ORs [89]. Hofer et al., [90] first described the expression of α-gustducin protein in GIT of rats which suggested the taste perception-related activity in other bodily organs. Like ORs, no special expression of any type of TRs have been studied in aquatic or terrestrial animals. However, TRs have been categorized into six types for showing unique expression channels and mediators. Currently, sour, sweet, umami, salty, bitter, and fat-sensing receptors are known to exist [88]. However, salt and sour TRs are sensed by membrane ion channels whereas, remaining are sensed with GPCRs [91]. Umami and sweet GPCRs are heteromers detecting wide range of amino acids [92] and belongs to family 1 (TAS1Rs).

### 3.1. Distribution of Odorant and Taste Receptors

The genomic analysis revealed existence varying number of OR genes in different species [93] and specie’s environment also effect the number of functional genes [94]. Almost 4% of mammalian genes are devoted to encode ORs [95] and in human around 400 functional genes of ORs are expressed at mRNA level [5,80]. Moreover, all human chromosomes harbor clusters of ORs genes except the Y and 20 chromosomes [80]. Similarly, all the mouse chromosomes have loci for ORs except 18 and Y chromosomes [96]. About 1000 ORs in rodents are encoded by GPCR family for the olfaction [86,97,98]. The number and distribution of OR genes indicated that higher primates rely mostly on their vision instead of chemical identification of environment via olfaction [99]. During the evolution process the gene gain and loss occurred in several species according to their adaption of the new environment [100]. Today’s ORs gene family has evolved after a course of birth-and-death evolution during which many genes were duplicated, and others become pseudogenes [101]. The pseudogenes resulted from nonsense-mutation, deletion, frameshift, and or combination of these [87]. The number of intact and pseudogenes of OR and TR families are shown in Table 2. No doubt, the functional differences among these receptors do exist but they all belong to the same GPCR protein family. 

Many studies have reported the presence of odorant receptor transcripts in heart, brain, germ cells, sperms, and mammalian testes [9,11,13,86,112]. Their essential physiological roles include skin wound repair [113], inhibition of proliferation of oncogenic cells in liver, prostate and intestine [8,114,115], and modulation sperm motility [7]. Despite of all the efforts made to unveil the functional specificity of these receptors in the non-olfactory tissues, only few ORs could be aligned with their ligands. More strenuous work is demanded to complete the profiling of these receptors in the reproductive organs.

Similar to ORs, TRs discovery in endocrine cells of mouse [116] led to the subsequent findings in other organs of the body. Recent exploration for TRs in ectopic tissues have resulted in localization in brain [117,118], kidney [119], respiratory system [91,120], cardiovascular system [121], immune system [122,123,124], skin [125], thyroid gland [126], urethra [127], testis [15,128], prostate and ovary [129], and adipose tissues [130,131]. These organs harbor different types of TRs, i.e., sweet, free fatty acids, bitter, and sour taste receptors. 

Although TRs have been extensively studied in regulation of appetite [132,133], diabetes [134], asthma [135], and other inflammatory processes [136,137] in the body but only little is known about reproductive TRs. TRs are being targeted by most of the available drugs used as therapeutic agents which indicates their potential role in the body. For example, TRs control the production of endocrine hormones from gut in response to volume and composition of food [138,139]. GI tract lining shows expression of carbohydrate, fatty acids, amino acids, and toxins sensing TRs (TAS1R1 to TAS1R3) if there are any in the feed [140,141,142]. Similarly, adipogenesis dependence on TRs have been identified through the expression of α-gustducin and TAS2Rs in pre-adipocytes and adipose tissues in mouse [91]. Similarly, free fatty acid TRs abundance in European people suggests their role in development of obesity due to mutations in taste responding genes [143]. Moreover, innate immunity and bronchodilation is governed by bitter taste receptors as they are expressed in immune cells, smooth muscles, and lung epithelial cells [144]. For instance, human nasal cilia express TAS2R38 which upon activation causes increase in Ca^2+^ that is responsible for increased beat frequency of cilia to clear mucous [145].

### 3.2. Reproductive Odorant and Taste Receptors

Testicular and sperm ORs of various species including dog, human, chimpanzee, mouse, and rat have been identified and some of them successfully related to their ligands. The ORs related to several mammalian species along with different techniques which were used to identify them are summarized in Table 3.

To verify the role of ORs, studies are being conducted at transcript and protein levels. Parmentier et al., [5] discovered 3 and 21 mRNAs of ORs in dog and human testis, respectively. Within a decade, researchers belonging to different countries discovered the odorant receptor transcripts in testis of mouse, hamster and rat [12,13,146,147,148,149,150]. More than 80 cell types and tissues of different species have been explored by using next generation sequencing and at least one OR was expressed in every sample. However, the number of odorant receptors expressed differed in different studies with highest in testes, heart, and brain, respectively [9,112,151,152]. This may be due to multiple screening approaches and inflexibility of the analysis. The ordinary nasal olfactory receptors contain less intact sequences of amino acids than testicular ORs which indicate an important physiological function of these receptors in testes [153]. Furthermore, by using high throughput oligonucleotide microarray, 66 genes equipped with odorant receptors were discovered in mouse olfactory epithelium [154].

**Figure 1:**
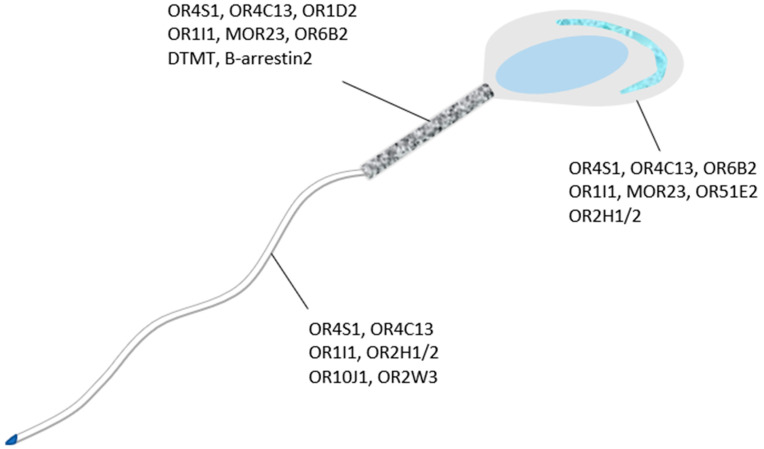
Different ORs/Protein localized on sperm head, mid-piece and tail. ORs in head of sperm cell are distributed at acrosomal, equatorial, and posterior head regions. Similarly, ORs of mid-piece and tail are also located along the entire lengths of these segments.

Oocyte and cumulus oophorous cells separately secrete odorants to attract the sperm [155]. The strong candidate for odorants of sperm like heparin, acetylcholine, and progesterone have been excluded from the list after experimental evaluation [39,156]. During in-vitro studies, it was proved that sperm cell demonstrate chemotaxis toward the chemicals withdrawn from follicular fluid (FF) [157]. The chemotaxis in human sperm was induced by the application of progesterone and RANTES (chemokines) which are the active ingredients of follicular fluid [158] which contradicts the aforementioned studies. Moreover, FF also contains heparin, Substance P, and β-endorphins which have proven to be substantial chemoattracts molecules for mouse, human, and pig sperm in vivo and in vitro [159,160,161]. Similarly, oocytes synthesizes more than ten types of prostaglandins which promote the sperm guidance and motility in the female reproductive tract [162,163]. Additionally, roles of estradiol, adrenaline, calcitonin, oxytocin, acetylcholine atrial natriuretic peptide, and nitric oxide have been verified up to some extent in sperm chemotaxis [39,164,165]. Furthermore, the sperm chemoattractant chemicals secreted by the oocytes and cumulus cells [155] are considered as a tool to attract only hyperactivated sperm toward the egg instead of attracting all the sperm in the female reproductive tract [166]. With the implementation of antibody staining techniques, odorant receptors were localized in head, tail, and mid-piece (Figure 1) of dog and other mammals’ spermatids [13,148]. However, due to unique amino acid sequence of testicular ORs they cannot be included in a specific odorant receptor subfamily [12].

Many studies have confirmed the expression and localization of TRs in mammalian testes and spermatic cells. Mammalian sperm cells undergo around 200 genes mutations which at any stage can hinder the process of spermiogenesis [178] and lead to sub-fertility and infertility. Xu et al., [15] have identified 35 bitter sensing genes in mouse testes that expresses post-meiotic sperm cells. This finding indicates potential role of TRs in successful development of sperm cells in male reproductive system and suggestive of their critical role in female reproductive system. From vagina to the site of fertilization, sperm cell senses slight variations in the macro and micro-environment including carbohydrates [179], amino acids [180,181,182], and changes in composition and pH [183,184]. Sperm detects chemo-attractants secreted by the egg and its surrounding environments in order to successfully fertilize the egg [4,20]. These chemo-attractants and sperm interaction were further consolidated by the work of Meyer et al., [185] who discovered umami taste receptor family dimers (TAS1R1 and TAS1R3) in sperms of human and mouse. This study determined the rate of acrosomal reaction, intracellular Ca^2+^concentration, and cAMP level along with the expression level of TAS1R1 which indicates their role in maturation of sperm in the female reproductive tract.

Other similar studies have confirmed the expression of sweet, umami, bitter, and fatty acid sensing receptors in mammalian testis [186,187,188,189,190]. Furthermore, Fehr et al., [191] discovered the expression of α-gustducin protein during spermatogenesis and GPCRs distribution along the flagellum of human, rat, mouse, and bull sperm cells. Additionally, Governini et al., [14] analyzed the expression of TAS2Rs family bitter taste receptors in human testis and ejaculated sperm which also consolidate the idea of clinical significance of TRs in human reproduction. Only handful of studies available regarding expression of TRs and related proteins in reproductive organs of mammals (Table 4).

## 4. Olfactory Transduction and Sperm Signaling Pathways

**Figure 2:**
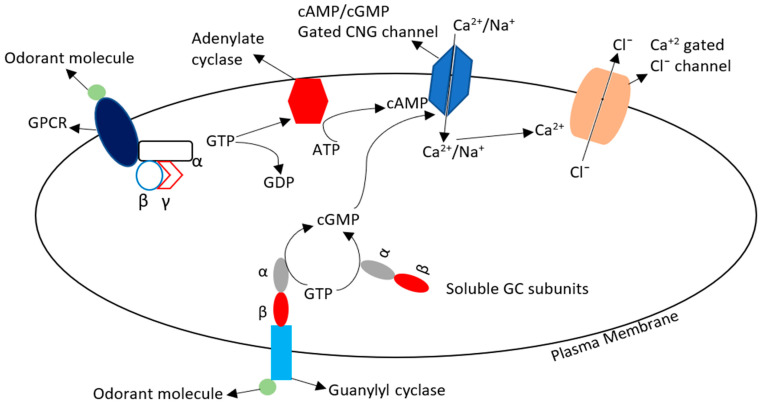
General mechanism of odorant receptor activation and signal transduction pathway. Odorant molecules combined with membrane GPCR and their cytosolic subunit α activates the adenylate cyclase (AC) by converting GTP into GDP. This AC mediates conversion of ATP into cAMP which causes opening of CNG channels. Similarly, when Odorant molecule interacts with (guanylyl cyclase) GC their cytosolic subunits convert the GTP to cGMP which enhances the opening of CNG channels. Ca^2+^ enters the cell through these CNG channels and increased Ca^2+^ concentration influences the opening of Ca^2+^ gated chloride (Cl^−^) channels. So, the influx of positive ion (Ca^2+^) and efflux of negative ion (Cl^−^) changes the membrane potential causing waves of polarization and depolarization which helps in sperm motility. GTP, guanosine triphosphate; GDP, guanosine monophosphate; ATP, adenosine triphosphate; cAMP, cyclic adenosine monophosphate; cGMP, cyclic guanosine monophosphate.

Binding of specific odorant molecule with its receptor initiates a cascade of reactions in the membrane and opens ion channels. The cyclic nucleotide gated Ca^2+^ ion channel is specific for mammals and is opened at higher concentration of cyclic adenosine monophosphate (cAMP) and cyclic guanosine monophosphate (cGMP). The olfactory neurons contain olfactory specific G-protein (Golf) which on stimulation causes activation of adenylate cyclase which in turn increases the level of cyclic AMP. While, the membrane-bound guanylyl cyclase (GC) responds directly to the odorant molecule binding and cytosolic soluble guanylyl cyclase converts GTP into cGMP [193]. This increased cAMP and cGMP causes increases in oxygen consumption [194] and opening of CNG channels which results in the influx of Ca^2+^ and Na^+^ and thus causes depolarization of the membrane. The Ca^2+^-gated Cl^−^ channels opening causes further amplification of depolarization and leads to generation of an action potential and hypermotility of sperm. This Cl^−^ efflux causes 50–90% change in membrane potential of olfactory sensory neurons (Figure 2). 

In past studies, it was proposed that the two classes of odorant receptors initiate two different signaling pathways including cAMP and IP3 [195,196,197,198]. Further it was proved that, IP3 pathway may play just a modulatory role [199], and olfactory signaling is solely mediated by cAMP pathway. However, a cross-talk between cAMP and tyrosine kinase has been determined which resulted in Ca^2+^ regulation and capacitation of sperm [200]. This interaction between moieties of these signaling pathways requires the presence of Ca^2+^ and HCO_3_^−^ and efflux of cholesterol from the cell [200,201]. Moreover, progesterone produced by the cumulus oophorous cells causes activation of CatSper channels which results in Ca^2+^ influx and hyperpolarization [202,203]. Bicarbonate (HCO_3_^−^) contents increases on mixing of epididymal and prostatic secretions which are essential for sperm hyperpolarization. The cAC, cAMP, and PKA pathways control the hyperactivity of sperm and are dependent on concentration of Ca^2+^ and HCO_3_^−^. The sAC is sperm specific and converts AMP into cAMP which results in the activation of serine/threonine kinase [204,205]. Other sperm signaling pathways include PIK3C-AKT and MAPK pathways of which the former act through the CatSper channels and causes influx of Ca^2+^. All these pathways result in phosphorylation of serine protein, a primary component of sperm motility [206]. cAMP appears to play a basic role in sperm motility via odorant receptors and activation and when mice were knocked out for cAMP pathway, they did not respond to any of the odorants [207,208,209]. 

## 5. Gustatory Transduction

**Figure 3:**
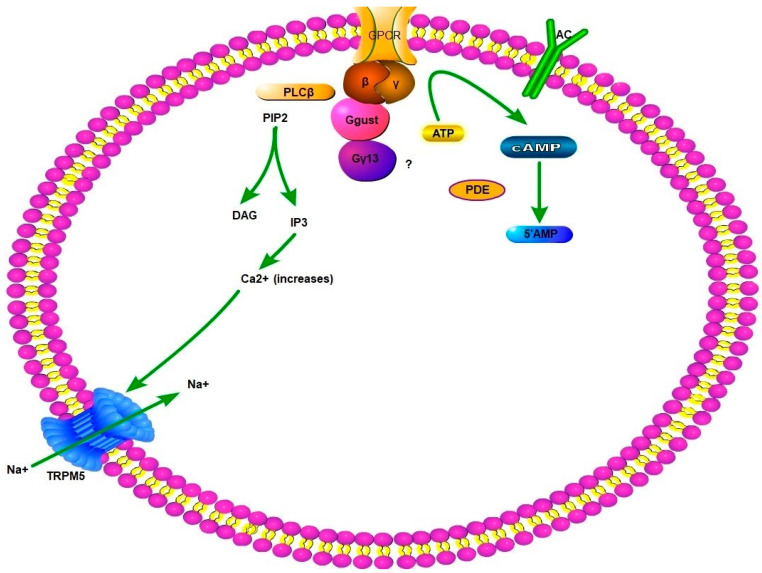
General mechanism of intracellular gustatory response. When tastant molecules combine with trans-membrane GPCR and the complex cytosolic subunit of βγ, Ggust, and Gγ13 cause conversion of ATP and PIP2 (with PLCβ, phospholipase Cβ) into cAMP and IP3, respectively. This IP3 mediated the increase of Ca^2+^ inside the cell through opening of channels in the Ca^2+^ storing organelles of cell, i.e., endoplasmic reticulum (not shown in the figure). This increased Ca^2+^ opens the TRPM5 channels which allow entry of Na^+^ ions. Both these positive ions change the membrane potential. Similarly, conversion of ATP into cAMP causes opening of AC channels which affects the downstream channels which may be similar to olfactory transduction signaling pathway. PLCβ, phospholipase Cβ; PIP2, phosphatidylinositol 4,5-bisphosphate; IP3, inositol 1,4,5-triphosphate; DAG, diacylglycerol; ATP, adenosine triphosphate; cAMP, cyclic adenosine monophosphate.

GPCRs involved in gustation possess many subunits including G-α transducing and gustducin, G-β3, G-γ13, and others. G-α and G-β mainly participate in taste perception and are closely linked in the cytosol. Upon interaction with a tastant molecule G-β subunit dissociates from G-α and initiate phospholipase-inositol activity which results in influx of Ca^2+^ in the cytosol [89]. This elevated Ca^2+^ results in the opening of TRPM5 (transient receptor potential cation channel subfamily M member 5) which depolarizes the taste perceiving cells [210]. Phosphodiesterase may be activated by G-α subunit, which leads to the breakdown of cAMP to avoid inhibition of phospholipase-inositol pathway [91,211]. The opened TRP channels lead to influx of Na^+^ and further depolarization due to opening of voltage-gated Na^+^ channels (Figure 3). These cellular processes cause release of ATP through Ca-homeostasis modulator 1 and 3 [212,213] which activates sensory neurons conveying messages to higher taste perception centers of brain. This taste perception or gustation has also been described in ectopic regions, but little is known about the expression of taste-related genes in reproductive system especially sperm. 

## 6. Cryopreservation and Olfactory Transduction

The cryopreservation elicits capacitation-like response in sperm i.e., increases intracellular Ca^2+^, pH, and cAMP along with plasma membrane and acrosomal membrane disintegration [214,215,216]. As we know, cryocapacitation-like changes after cryopreservation may largely contribute to lower sperm survival, motility, and fertility. It was reported that ORs, expressed in testes and mature sperm, are involved in sperm motility, sperm-egg recognition etc., [10,11,13,155]. Some specific odorant receptors in mid-piece and flagellum of sperm were found to be associated with spermatogenesis, epidydimal maturation, and sperm function [6,11]. Furthermore, some odorant-like molecules, including progesterone, heparin, ANP in the reproductive tract and oviduct can enhance or stimulate sperm chemotaxis or thermotaxis. These odorant-like molecules, probably combine with odorant receptors in sperm and increase intracellular Ca^2+^ level which results in rapid beat frequency of sperm flagellum and thus movement of sperm [19]. In frozen-thawed stallion sperm, the increased level of intracellular Na^+^ disturbed the membrane ion channels and mitochondrial membrane potential which indicate apoptotic-like changes [217]. Moreover, study showed lower cAMP concentration in frozen-thawed sperm as compared to fresh sperm in rat [218]. Cryopreservation negatively affects the cAMP-dependent PKA and AMPK activities in Atlantic salmon sperm [219]. These results indicate possible prior utilization of Ca^2+^ and Na^+^ due to activation of their membrane channels as a result of interaction with ligands during process of cryopreservation. In one study on pig sperm, when AMPK was inhibited, the sperm motility was also decreased [220]. Moreover, the human and boar frozen-thawed sperm have shown decreased response to progesterone compared to freshly ejaculated sperm, which lead to early depolarization and Ca^2+^ influx [221]. This Ca^2+^ influx is definitely related to activation of its voltage and ion gated channels which have not been proven yet. How these channels play role in Ca^2+^ transport and recognize chemical signals is an under-debate subject.

Recently, the report from high throughput sequencing in giant panda indicated that Ca^2+^-gated signaling, cAMP, cGMP, and neuroactive ligand receptor interaction pathways are enriched in post-thaw giant panda sperm [22]. It is demonstrated that giant panda sperm appears to have strong freeze tolerance capacity and is capable of surviving repeated freeze-thaw cycles [23]. These reports suggested that the cryo-resistance of sperm might be related to cAMP, cGMP, and Ca^2+^ abundance during cryopreservation and unique interaction between ligands and their receptors. More evidence showed that olfactory transduction pathway-related CNGB1 and CNGA3 channel proteins are enriched in post-thaw giant panda sperm. CNGA3 is a predicted target of three novel differentially expressed miRNAs (conservative_NW_003218630.1_359427, conservative_NW_003218630.1_359428, and unconservative_NW_003218488.1_343143). Therefore, the involvement of cAMP and Ca^2+^ may be responsible for regulating post-thaw quality of sperm [22]. Additionally, our ongoing studies have proved that cryopreservation causes m6A modifications in post-thaw sperm which are related to sperm cryo-injuries, freezability, and metabolic activities (unpublished work) along with altered expression of sperm olfactory genes.

## 7. Perspective

Odorant and taste receptors are extensively expressed in ectopic organs and their role in wound healing, cancer cells division inhibition, and obesity have been identified (19, 10, 26, 29). Targeting of TAS2R family and OLFR544 from ORs and TRs, respectively, shows promising future in controlling obesity and asthma [188,189,222]. However, mysteries regarding testicular and sperm odorant and taste receptors interaction and functionality still need to be revealed. Researchers are trying to determine the role of ORs and TRs in sperm-egg communication and in pre-fusion processes like capacitation induction, acrosomal reaction, and movement from uterine and oviductal storage. The role of these receptors in sperm chemotaxis and the role of egg, fallopian tube, uterus, and cervix in producing odorant [223] and tastant molecules need to be deciphered to maximize the success of artificial breeding.

Similarly, it is hypothesized that the components of cryoprotectants may act as ligands for the sperm ORs and elicit early capacitation which result in decreased motility, viability, and acrosomal integrity. Currently, no data are available on the topic of cryopreservation effects on modulation of gustation in sperm physiological parameters. But, the low survivability of pig’s sperm in comparison to other mammalian species indicate involvement of some hidden entities including ORs [22,224] and TRs.

Researchers opine that individual sperm may express distinct set of ORs and TRs and respond differently as speculated for odorant sensory neuron’s “one neuron-one receptor” model [225,226]. If this is true for sperm than the complexity of behavioral cues will require further exploration. Moreover, the opening of ion channels which are involved in olfactory and gustatory transductions i.e., CatSper1, CatSper2, and CNGA3 (Ca^2+^ channels) require different concentration of cAMP and cGMP [68,70,71,227] which has not been determined yet for all the mammalian sperm. 

In conclusion, ORs and TRs are associated with spermatogenesis, maturation, sperm-egg recognition, sperm chemotaxis, capacitation, motility, and fertility. In addition, ORs and olfactory transduction pathway were thought to be responsible for sperm freeze tolerance and post-thaw sperm quality. However, what and how many odorant-like molecules in the extenders and reproductive tracts interact with ORs to facilitate sperm chemotaxis, capacitation, hyperactivation, and fertility, are still unknown and worth further studying.

## Figures and Tables

**Table 1:** Ion and voltage-gated channels of mammalian sperm.

Location	Specie				
	Human	Bovine	Dog	Mouse	Rat
**Head**	Ca_v_2.1^c^, Ca_v_3.1^d^, Ca_v_3.3^d^, TRPC1^a^, TRPC3^a^, TRPC4^a^, TRPC6^a^, IP3R ^j^	IP_3_R ^j^	IP_3_R ^j^	Ca_v_1.2^b^, Ca_v_2.1^c^, Ca_v_2.2^d^, Ca_v_2.3^d^, Ca_v_3.1^d^, Ca_v_3.2^d^, TRPC2^a^, IP_3_R ^j^	IP_3_R ^j^
**Mid-piece**	Ca_v_1.2^b^, Ca_v_2.3^d^, Ca_v_3.1^d^, Ca_v_3.2^d^, Ca_v_3.3^d^, TRPC3^a^, TRPC4^a^, TRPC6^a^	CNGA3^e^		Catsper2^h^, Ca_v_2.1^c^, Ca_v_2.3^d^, Ca_v_3.1^d^, Ca_v_3.3^d^, TRPC1^a^, TRPC3^a^, TRPC6^a^	
**Upper tail**	Ca_v_1.2^b^, Ca_v_3.1^d^, Ca_v_3.2^d^, TRPC1^a^, TRPC4^a^, TRPC6^a^	CNGA3^e^, CNGB1^f^		Catsper1^g^, Catsper2^h^, Ca_v_1.2^b^, Ca_v_2.2^d^, Ca_v_2.3^d^, Ca_v_3.1^k^, Ca_v_3.2^d^, Ca_v_3.3^d^, TRPC1^a^, TRPC3^a^	
**Lower tail**	Ca_v_2.3^d^	CNGA3^e^		Ca_v_3.2^d^, TRPC3^a^	

**Note:** TRPC, transient receptor potential channel; Ca_v_, voltage-gated calcium channels; Catsper, cation channel of sperm; IP**_3_**R, inositol tri-phosphate receptor; CNG, cyclic nucleotide gated (Channels). ^a^ [58,59,60,61]; ^b^ [62]; ^c^ [63]; ^d^ [64,65,66]; ^e^ [67,68]; ^f^ [69]; ^g^ [70]; ^h^ [71]; ^j^ [72]; ^k^ [73].

**Table 2:** The number of odorant and taste receptor genes in different species.

Specie	Olfactory Genes Distribution		Reference	Taste Genes distribution		Reference
	Intact genes	Pseudogenes		Intact genes	Pseudogenes	
**Human**	396	425	[99]	38 (T2R)	16 (T2R)	[102,103]
**Chimpanzee**	380	414	[104]	28 (T2R)	10 (T2R)	[105,106]
**Cow**	1186	1057	[87]	12 (T2R)	15 (T2R)	[107]
**Dog**	811	278	[100]	15 (T2R)	5 (T2R)	[107]
**Horse**	1066	1569	[87]	19 (T2R)	36 (T2R)	[108]
**Mouse**	1130	236	[87]	35 (T2R)	5 (T2R)	[102]
**Rat**	1207	508	[100]	36 (T2R)	7 (T2R)	[109]
**Rabbit**	768	256	[87]	28 (T2R)	13 (T2R)	[110]
**Pig**	1113	188	[111]	15 (T2R)	7 (T2R)	[110]

**Table 3:** Mammalian reproductive odorant receptors/proteins.

Species	Organ	Receptors	Analysis Method	Reference
**Human**	**Sperm**	OR7E24, OR4S1, OR4C13, OR1I1, HT2, OR1D4, OR51E1, OR51E2, OR6B2, OR10J1, OR2H1/2, OR2W3	Confocal microscopy, WB, ICS	[10,167,168]
	**Prostate**	OR51E2, o1r59, Olfr78,	RT-PCR, WB, NB	[8,169]
	**Testis**	OR4C13, OR7A5, OR4D1, OR1D2, OR1D2, OR4D1, OR1D2, OR1E1 hOR17-4, hOR17-2, Olfr16, OR4N4, OR3A2, OR10J1	RT-PCR, q-PCR, Ca-Imaging, RNA-seq	[6,9,10,170]
	**Placenta**	hOR-17	RT-PCR	[171]
**Mouse**	**Sperm**	MOR23	RT-PCR, in situ hybridization	[11]
	**Prostate**	N/A	N/A	N/A
	**Testis**	MOR23, MOR244-3, MOR139-3, MOR248-11, MOR267-13, MOR283-1, MOR8-1, MOR31-2, OR10J5,	RT-PCR, NB	[11,12,172,173]
	**Placenta**	Olfr154, Olfr520, Olfr433, O1fr381	Microarray	[174]
**Dog**	**Sperm**	DTMT	RNase protection assay, WB	[44,148]
	**Prostate**	N/A	N/A	N/A
	**Testis**	DTPCR64, HGMP07, DTMT, OR1E2,	RT-PCR	[5,12]
	**Placenta**	cOR2AV3	Microarray, RT-PCR	[175]
**Rat**	**Sperm**	Putative olfactory Proteins GRK3, beta-arrestin2, G*olf*	WB, ICC, IH	[13,176]
	**Prostate**	N/A	N/A	N/A
	**Testis**	OD1, OD2, Olr825, Olr1696	RT-PCR, WB, IH, ISH	[13,177]
	**Placenta**	O1r1767, Olr1513, Olr1687, Olr1571	RT-PCR	[171]

**Note:** WB, Western blotting; NB, Northern blotting; RT-PCR, reverse transcription polymerase chain reaction; q-PCR, quantitative polymerase chain reaction; RNA-seq, RNA sequencing; Ca-imaging, calcium imaging; IHC, immunohistochemistry; ICC, immunocytochemistry; ISH, in situ hybridization; ICS, immunocytochemical staining; N/A, data are not available.

**Table 4:** Mammalian reproductive taste receptors/proteins.

Specie	Location	Receptors/Protein	Analysis Method	Reference
Human	Sperm	Tas1r1, Tas1r3, α-gustducin, α-transducin	RT-PCR, WB	[14,185]
	Testis	Tas1r14	Droplets digital PCR	[14]
Mouse	Testis	Tas2r102, Tas2r 105, Tas2r 106, Tas2r 113, Tas2r 114, Tas2r 116, Tas2r 124, Tas2r 125, Tas2r 134, Tas2r 135, Tas2r 136	RT-PCR	[15]
Boar	Sperm	α-gustducin, α-transducin	WB, immunohistochemistry	[16]
	Testis	T1R3	RT-PCR, in situ hybridization	[192]

## Data Availability

Not applicable.
